# Automatic Structural Parcellation of Mouse Brain MRI Using Multi-Atlas Label Fusion

**DOI:** 10.1371/journal.pone.0086576

**Published:** 2014-01-27

**Authors:** Da Ma, Manuel J. Cardoso, Marc Modat, Nick Powell, Jack Wells, Holly Holmes, Frances Wiseman, Victor Tybulewicz, Elizabeth Fisher, Mark F. Lythgoe, Sébastien Ourselin

**Affiliations:** 1 Centre for Medical Imaging Computing, University College London, London, England, United Kingdom; 2 Centre for Advanced Biomedical Imaging, Division of Medicine, University College London, London, England, United Kingdom; 3 Department of Neurodegenerative Disease, Institute of Neurology, University College London, London, England, United Kingdom; 4 Division of Immune Cell Biology, MRC National Institute for Medical Research, London, England, United Kingdom; INSERM, Paris, France

## Abstract

Multi-atlas segmentation propagation has evolved quickly in recent years, becoming a state-of-the-art methodology for automatic parcellation of structural images. However, few studies have applied these methods to preclinical research. In this study, we present a fully automatic framework for mouse brain MRI structural parcellation using multi-atlas segmentation propagation. The framework adopts the similarity and truth estimation for propagated segmentations (STEPS) algorithm, which utilises a locally normalised cross correlation similarity metric for atlas selection and an extended simultaneous truth and performance level estimation (STAPLE) framework for multi-label fusion. The segmentation accuracy of the multi-atlas framework was evaluated using publicly available mouse brain atlas databases with pre-segmented manually labelled anatomical structures as the gold standard, and optimised parameters were obtained for the STEPS algorithm in the label fusion to achieve the best segmentation accuracy. We showed that our multi-atlas framework resulted in significantly higher segmentation accuracy compared to single-atlas based segmentation, as well as to the original STAPLE framework.

## Introduction

Genetically modified mice are widely used in the preclinical studies of human brain diseases such as Alzheimer’s disease, as they share more than 85% of their genes with humans [1]. Since an estimated 20,000 knockout mouse strains will be created by the International Knockout Mouse Consortium over the coming decade [2], advanced computational imaging tools will be essential for efficiently extracting information. Worldwide efforts to understand the role of genes in brain morphology demand efficient data acquisition and analysis framework to quantify the consequences of gene function in development and pathology.

High resolution MRI techniques (voxel size <100 µm) are becoming an increasingly popular tool to study morphometric changes in transgenic mice. Large scale MRI phenotyping studies demand high-throughput acquisition and analysis of high-resolution 3D data. In particular, automatic, accurate quantitative methods for MR image analysis are essential for effective phenotyping. Structural parcellation is a quantitative analysis method, which enables the morphometric characterisation of brain structures, such as shape and volume. The current gold standard for structural parcellation in MRI studies of mouse brains is conducted manually, despite being expert-dependent and labour intensive [3], [4]. Different automatic algorithms have thus been developed to overcome these limitations and meet the challenge of objective and accurate high throughput analysis [5], [6].

Segmentation propagation is a method for automatic structural parcellation [7]–[10]. It uses pre-labelled MR images – called “atlases” – to automatically segment different anatomical regions of an unlabelled MR image. Here we define an atlas as a pair of images containing both the original MR data and its corresponding manually labelled anatomical structures. Firstly, a transformation is performed which maps the original MR data of the atlas to the unlabelled MRI in a process called image registration. Secondly, the same transformation is applied to the manually labelled anatomical structures in order to match the unlabelled image’s morphology. The performance of the segmentation propagation method relies highly on the image registration procedure [11]. Local misalignments can occur due to the large morphological variability between subjects, imaging artefacts, low signal/contrast-to-noise ratios and different contrasts (i.e. T1, T2, T2* weighted), resulting in poorly propagated segmentations.

Several studies have tried to improve the accuracy of segmentation propagation methodologies by propagating several atlases’ labels and then merging them into a more accurate result; this concept is known as “label fusion” [12], [13]. Aljabar et al. have shown that segmentation accuracy is dependent on the number of atlases used [14]. They tested label fusion using a majority voting strategy, in which each voxel is assigned to the structural label upon which the majority of the propagated atlases agree. They concluded that the segmentation accuracy reaches a maximum value when the number of atlas selected for fusion reaches a certain number.

Several methods have been introduced to achieve automatic structural brain parcellation in clinical MRI studies [8], [15]–[17], especially using the multi-atlas based approach [12], [18]–[24]. However, only a handful of studies have applied multi-atlas based structural parcellation techniques to preclinical data. Artaechevarria et al. segmented *ex vivo* mouse brains using a multi-atlas approach [25]. Their study relied on an *ex vivo* mouse brain MRI atlas database with 10 individual samples that had each been manually segmented into 20 structural labels [26]. In their study, a weighted majority voting label fusion method was used, with the weights derived from the mutual information between the two registered images. They showed improvement in segmentation accuracy when compared to a simple majority voting method. In the case where only one atlas is available, Chakravarty et al. [27] proposed a method which firstly propagates the atlas labels to a set of unlabelled images using a conventional single-atlas segmentation propagation approach. Subsequently the resulting set of structural labels were propagated to the target image using majority voting, demonstrating improvements in terms of segmentation accuracy when compared with direct single-atlas segmentation propagation.

The above-mentioned preclinical studies are largely dominated by *ex vivo* data sets. Recently, there has been a shift from *ex vivo* towards *in vivo* MRI phenotyping, limiting artefacts from tissue preparation and enabling longitudinal studies [3], [28], [29]. However, *in vivo* studies inevitably generate images with much lower contrast/signal-to-noise ratio due to the shortened scanning time and the limited use of contrast agents. There is a current need for robust methodologies to process these data. Scheenstra et al. proposed an automatic structural parcellation of *in vivo* mouse brain MR images by firstly performing a single-atlas affine registration-based segmentation, followed by an edge-based clustering in order to achieve a fast segmentation [30]. This method is shown to achieve the same level of segmentation accuracy compared to non-rigid registration. More recently, Bai et al. [5] conducted a study to compare structural parcellation accuracy using various methods and found no significant improvement when using a more advanced label fusion algorithm, the simultaneous truth and performance level estimation (STAPLE) [12], [13], or a Markov random field approach alone, compared to a simple majority voting approach.

Cardoso et al. have recently proposed a multi-atlas label fusion algorithm, the multi-label similarity and truth estimation for propagated segmentations (STEPS) [21], which integrated the Markov random field regularisation into the optimisation scheme in the STAPLE framework, along with some other improvements, including a spatially variant atlas ranking scheme based on the locally normalised cross correlation (LNCC). Validation performed on human brain MRI data showed better segmentation accuracy using STEPS compared to other multi-atlas label fusion algorithms.

In this paper, we apply a fully automatic multi-atlas based open source framework to the structural parcellation of mouse brain MRI. The framework consist of several preprocessing steps along with a non-rigid B-spline parameterised registration [31], and the above mentioned label fusion method STEPS. We investigated the parameters in the STEPS label fusion algorithm, and optimised those parameters for the *in vivo* mouse brain atlas database, the MRM Neurological Atlas (MRM NeAt), provided by Ma et al. [3]. We then evaluated the performance of our framework by comparing it with a single-atlas based method without any label fusion technique as well as with the commonly used STAPLE label fusion algorithm. We also demonstrated the ability of our framework to parcellate new unlabelled images by adopting another *in vivo* mouse brain MRI atlas, the National University of Singapore (NUS) mouse atlas [5] and regarded the MR images in it as unlabelled test images. We further tested the ability of our framework to detect volumetric difference between brain structures of mice with or without genetic modification.

## Materials and Methods

In this section, we firstly introduce the multi-atlas framework of the automatic structural parcellation step by step. Secondly, we describe the *in vivo* mouse brain atlas which we use for evaluation. Thirdly, we present the optimisation of the STEPS algorithm and evaluated its performance using the *in vivo* atlas. Fourthly, we apply and evaluate the ability of our framework to parcellate new unlabelled *in vivo* MRI data. Finally, we evaluated the ability for groupwise analysis using our framework on a previously published *ex vivo* MRI dataset.

### Automatic Multi-atlas Structural Parcellation Framework Construction

**Figure 1:**
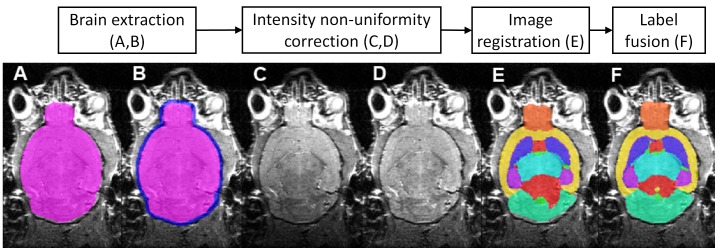
Step-wise summary of the framework. Pipeline of the framework is shown at the top of the image. Below the pipeline are representative images of results obtained after each processing step of the framework when applied to an unlabelled image. (A) Brain extraction – create brain mask for bias field correction; (B) Dilate mask to include contrast of brain tissues and CSF for image registration; (C, D) Images before and after bias field correction; (E) Structural parcellation result after single-atlas segmentation propagation; (F) Structural parcellation result after multi-atlas label fusion.

The automatic multi-atlas framework includes two pre-processing steps (brain extraction and bias field correction) followed by a series of non-rigid registrations and a final label fusion step. Figure 1 shows a step-by-step summary of the pipeline.

#### 1. Brain extraction

Brain extraction is an important pre-processing step to limit the analysis region of interest (ROI) to areas specifically within the brain region. In this step, a mask is created for the unlabelled image that includes only the regions containing brain tissues and excludes all other non-brain tissues and background. The mask of the unlabelled image is created automatically from the atlas images through the following steps. Firstly, the unlabelled image is globally registered to all atlas images, with the cost function in the optimisation step calculated over only the voxels inside the atlas mask and their corresponding voxels in the warped unlabelled image. This global registration steps are performed using a block-matching approach [32]. Secondly, the resulting transformation matrices are inverted and used to propagate all the atlas brain masks to the unlabelled image. Thirdly, all the propagated brain masks propagated from the atlas database are fused using the STAPLE algorithm [12] in order to obtain a consensus brain outline. Finally, the mask is dilated by 4 voxels so that the contrast between brain tissue and the surrounding CSF can be captured by non-rigid image registration in a later step.

#### 2. Intensity non-uniformity correction

MR images are corrupted by intensity non-uniformity, caused by factors such as the inhomogeneity of the RF excitation field and the spatially non-uniform distributed receiver coil sensitivity profiles [33]. The corrupted intensity profiles may lead to misalignment in the registration process. To correct this problem, we adopted the N3 intensity non-uniformity correction algorithm developed by Sled et al. implemented in FreeSurfer [33]. The characteristic distance over which the field varies was set to 10 mm, with a deconvolution kernel used to sharpen the histogram set to 0.15 mm, a threshold of percentage change in field estimate, below which iteration stops, set to 0.0001 and a maximum iteration number set to 100.

#### 3. Image registration

After the intensity non-uniformity correction was implemented, the affinely aligned atlas images obtained in the brain extraction step were then non-rigidly registered to the unlabelled image. The non-rigid registration aims at maximising the normalised mutual information using a cubic B-spline parameterisation to model the transformation [31]. The transformations obtained from the above registration procedures were then used to warp the manually segmented structural labels of the atlas images into the space of the unlabelled image. Nearest-neighbour interpolation was used to preserve the integer nature of the labels.

#### 4. Label fusion

After all the propagated structural labels were obtained from the image registration step, they were fused to generate the final result of structural labels for the unlabelled image. We adopted the STEPS algorithm developed by Cardoso et al. [21] to perform the label fusion. STEPS is an extension of the original STAPLE algorithm proposed by Warfield et al. [12], and extended by Rohlfing et al. [13]. The original STAPLE algorithm was developed with the purpose of fusing several expert-delineated manually labelled anatomical structures in order to obtain the hidden ground truth segmentation. Several improvements over STAPLE were introduced in STEPS. A more detailed derivation of the STEPS algorithm is described in Cardoso et al., 2013 [21]. In the original paper, Cardoso et al. have compared the STEPS algorithm to several other label fusion methods, and demonstrated that STEPS resulted in the highest parcellation accuracy under the same setting, and was most robust to the reduction of database size. Representative resulting images obtained after each processing step of the framework when applying to an unlabelled image are shown in Figure 1.

### Mouse Brain Atlas

To optimise the parameters for label fusion and evaluate the accuracy of the parcellation results, we adopted the publicly available mouse brain MRI atlas databases. Currently, the number of such available atlas databases is limited. To the best of our knowledge, there are currently 7 publicly available atlas databases [3], [5], [26], [34]–[40], most of which contain only one structurally labelled average atlas (the minimal deformation atlas). A detailed comparison of all the databases is presented in the supplementary material “File S2. MRI mouse brain atlas databases currently available”. Within the databases, only two of them, the MRM NeAt [3], [26] and the NUS atlas [5], contain structural labels for each individual atlas sample, which make it possible to be adopted by the proposed multi-atlas based label fusion method. Both of these two atlas databases include *in vivo* image samples, and the brain structures were both manually parcellated following the Franklin-Paxinos atlas [41]. The MRM NeAt database includes atlases of 12 individual T2*-weighted *in vivo* brain MR images of 12–14 weeks old C57BL/6J mice; each with 20 manually labelled anatomical structures. The NUS mouse atlas database includes 5 individual T2-weighted *in vivo* brain MR images of adult male C57BL/6J mice, each has 40 manual labelled anatomical structures. Detailed scanning parameters are described in Ma et al., 2008 [3] and Bai et al. 2012 [5]. Heckemann et al had previously shown that increasing the number of images in the atlas database can improve the accuracy of the label fusion derived consensus segmentation [8]. As a result, the MRM NeAt atlas database, which has the largest number of atlases, was selected for this part of the study. Due to missing labels in 2 of the 12 available atlases in the MRM NeAt database, only 10 images and associated structural labels were included.

For neurodegenerative diseases, the progression of pathology might vary between two hemispheres [20]. Furthermore, for studies interested in further estimating the cortical thickness from the structural parcellation result, hemisphere separation can also help to identify and segment the intra-hemispheric cortical surface area [42], [43]. It is thus preferable to separate the structural labels of the original atlas into left and right hemispheres. We thus separated the brain images and their corresponding structural labels in the original atlas database into left and right hemispheres along the mid-sagittal plane. Maes et al. achieved left/right hemisphere separation for asymmetry measurement using segmentation propagation [44]. An alternative way to determine the inter-hemisphere separation plane is to exploit the symmetric nature of the MR data. This method aims at finding the reflective rigid-body transformation that minimizes the absolute distance of an image and its mirrored version [45]–[47]. This method is only valid for brain images from wild type mouse strains, for which no left/right asymmetries are induced by diseases. Since the mice in the atlas database adopted in this study are wild type animals, we used the latter method by firstly flipping the atlas images and using normalised mutual information as an asymmetry measurement to find the mid-sagittal plane.

### Parameter Optimisation

In the STEPS algorithm, the best local labels for label fusion are selected after ranking based on the LNCC computed over a local Gaussian kernel [21]. As a result, the segmentation accuracy varies depending on two user-specified parameters. The first is the width of the Gaussian kernel used to estimate the LNCC for locally ranking the propagated atlases. The second is the number of top ranked atlases to include in the local label fusion. In this paper, we optimised the parameters on the MRM NeAt atlas database after the labels have been separated into left and right hemisphere. We varied the Gaussian kernel standard deviation from 1 to 6 voxels (incremental step of 0.5 voxel) and the number of atlases used from 3 to 9. In total, 77 parameter combinations were calculated.

For each pair of parameters, we calculated the average Dice similarity coefficient of every atlas across the entire database as an indicator of the structural parcellation performance. The average Dice similarity coefficient is obtained in the following steps (known as a “leave one out cross validation”). Firstly, each of the 10 images was regarded as an unlabelled test image, and the remaining 9 were used as the atlas images. The structural labels in the atlases were propagated to the unlabelled image with multi-atlas segmentation propagation scheme. Secondly, the Dice similarity coefficients between the automatic segmentations and the manual segmentations were calculated for every image in the database. Finally, the averaged Dice similarity coefficients for all the images across all structures were calculated for each parameter combination. The combination that gave the highest average Dice similarity coefficient was selected and regarded as the optimal set of parameters.

### Performance Evaluation

In order to evaluate the performance of the multi-atlas label fusion part of our framework, we compared it with a single-atlas based segmentation propagation method as well as with the commonly used multi-atlas label fusion method STAPLE. A leave-one-out cross validation similar to what was described in the parameter optimisation section was performed for both methods. For the single-atlas method, we propagated the label from each of the 9 atlases and averaged the Dice similarity coefficient.

### Application to Unseen Images

MR images collected from different sites and studies may vary due to various factors such as scanner/coil variance and scanning sequence differences. As a result, in addition to the cross validation within the same atlas database, we tested the performance of our framework to parcellate images collected from sites other than the atlas database, to further evaluate its performance in the situation of a real application.

To quantitatively evaluate the performance of our multi-atlas framework when applied to a new dataset, an expert-delineated manual structural parcellation is required for the new dataset as a gold standard. Here we use the atlases in the NUS atlas database [5] as unlabelled test images. We separated the corresponding manual structural labels into left/right hemisphere as we did for the MRM NeAt atlas database, and generated 40 structural labels for each hemisphere.

We selected and grouped 24 structural labels (12 in each hemisphere), which were presented in the manual segmentation in both of these two atlas databases to ensure a one-to-one structural correspondence between all atlases. However, the inter-rater variability still needs to be taken into account. This is due to the differences in the manual structural parcellation protocols between two databases, and the subsequent accuracy of the quantitative analysis [48]. For the two atlas databases we adopted, the manual structural parcellations were both following the Franklin-Paxinos atlas [41]. Nevertheless, giving the fact that there is no knowledge about the inter-rater variability between these two datasets, there is still a source of variability in the experiment. This limitation is discussed in more detail in the “conclusion and discussion” section.

Similarly to the procedure described in the performance evaluation step, we propagated the structural labels from the MRM NeAt database to each of the unlabelled images from the NUS database using our multi-atlas framework, as well as the STAPLE algorithm and single-atlas segmentation propagation method. We adopt the parameters we previously obtained to fully represent a real situation, where no manual segmentation are available to optimise the parameters. Finally, for each of the three approaches, we calculated the Dice similarity coefficient between the automatic parcellation results and the manual segmentations in the NUS database, and compared the results.

### Application to the Groupwise Analysis

One of the main applications of structural parcellation is to detect and quantify volumetric changes in brain structures of different animal groups, which vary in terms of pathology or genetic background. To test the ability of our framework to detect such statistical differences, we adopted previously published data set of the Tc1 mouse model of Down syndrome. Details about the Tc1 model and image acquisition are described in Sinclair et al. [49]. *Ex vivo* mouse brains of 16 animals, 8 wild type and 8 transchromosomic, were selected and structural parcellated. The MRM NeAt database also includes 10 *ex vivo* atlas images, which were manually parcellated into the same 20 structures [26]. We thus adopted these *ex vivo* atlases, again with structural labels separated into left and right hemisphere (one of these 10 *ex vivo* atlases is discarded due to the artefact as well as its resolution difference compared with other *ex vivo* atlases in the database). Similarly to the *in vivo* database, we used a leave-one-out cross-validation strategy to obtain an optimised combination of parameters using the atlases. We then applied the proposed framework to parcellate the structures of all 16 animals. Both our framework and the single-atlas method were used to detect volume differences in all structures between the wild-type group and the transchromosomic group. The obtained volumes were compared both with and without total intracranial volume normalisation.

## Results

We firstly optimised the parameters of the STEPS algorithm in our framework, and evaluated its performance using leave-one-out cross validation for the *in vivo* atlas database MRM NeAt. Secondly, we compared the segmentation accuracy obtained from our pipeline with the result obtained from a single-atlas segmentation propagation method, and with the STAPLE algorithm. Thirdly, we adopted the *in vivo* mouse brain MRI data from another atlas database, the NUS mouse atlas, as test images, and validated the ability of our multi-atlas framework to parcellate unlabelled new data from different site. Finally, we applied our framework on *ex vivo* MRI data from two groups of mice with different genetic background to evaluate the ability to detect volumetric difference between groups. The framework source code and a sample atlas database can be downloaded from http://cmic.cs.ucl.ac.uk/staff/da_ma/multi_atlas/.

### Parameter Optimisation

**Figure 2:**
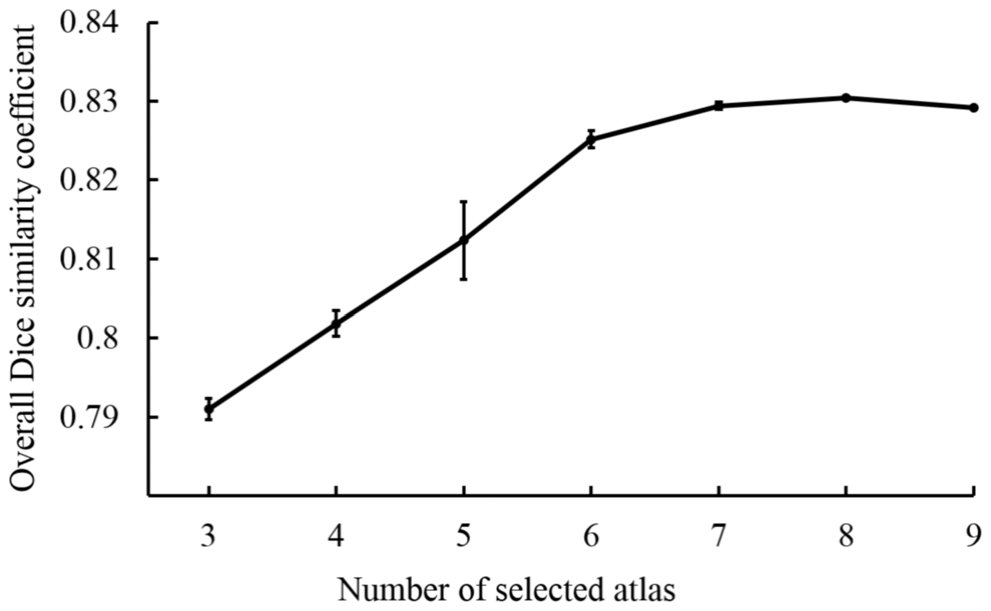
Parameter optimisation for atlas database with left/right hemisphere separated. The overall Dice similarity coefficient across all structures resulted from the selection of different number (from 3 to 9) of top-ranked atlases for label fusion. The error bars represent the standard deviation of 12 tests with different Gaussian kernel standard deviation in the LNCC image similarity measurement (from 1 to 6 with 0.5 step increment). The small variation indicates little effect of the Gaussian kernel width towards the overall accuracy.

Optimal parameters were obtained for the MRM NeAt atlas database. We optimised the values of two parameters in the local ranking system LNCC of the STEPS algorithm: the width of the Gaussian kernel for image comparison and the number of top ranked labels to include in the label fusion. Between the two parameters, the number of top-ranked atlas selected for label fusion appears to have a dominant effect on the performance. Figure 2 shows the Dice similarity coefficient value obtained with respect to different number of atlases selected, where the error bars represent the variation caused by selecting different values of the Gaussian kernel standard deviation in the LNCC image similarity measurement. The segmentation accuracy was estimated as the average Dice similarity coefficient across all the structures, which varies from 0.79 to 0.83.

**Figure 3:**
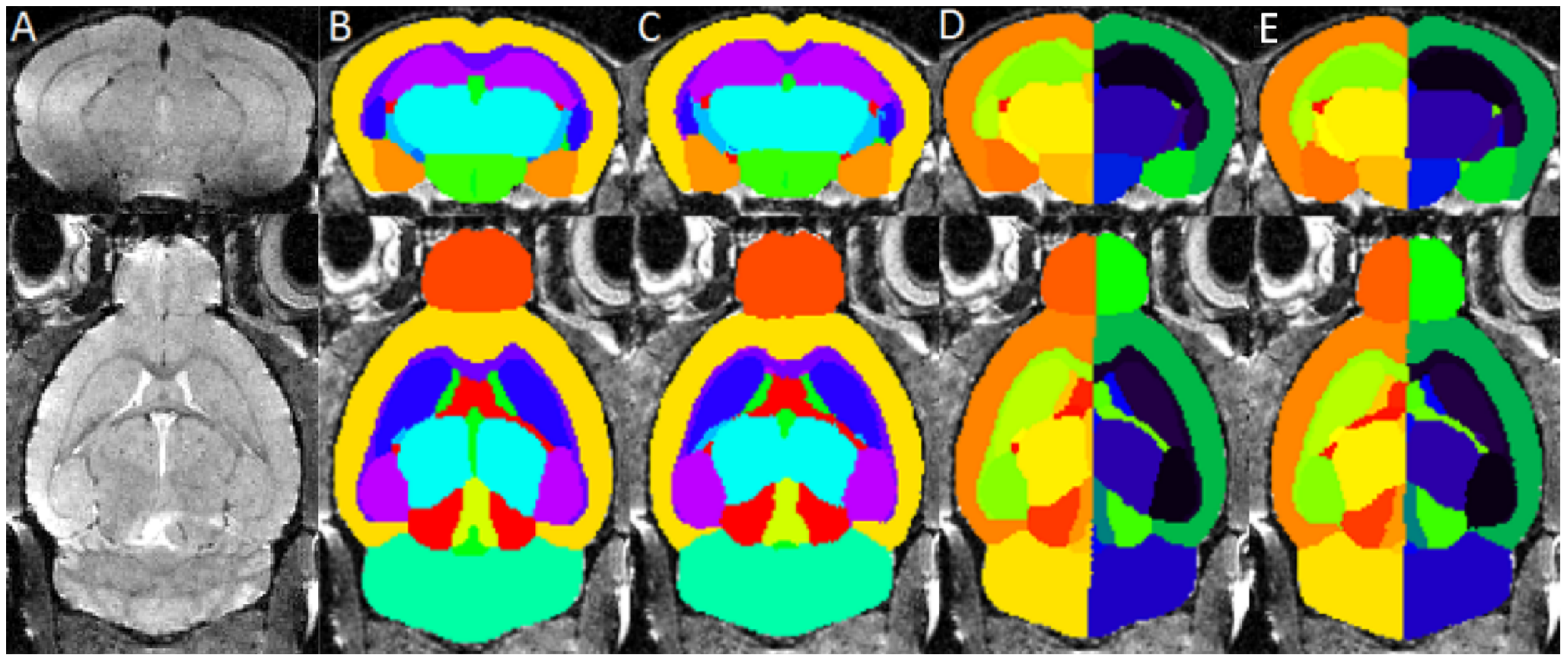
Sample images from the cross validation result of the pipeline on the atlas databases. Parcellation results obtained with the proposed method and parameters. (A) The original MR image from the atlas (B, D) The MR image from the atlas overlaid with corresponding manually labelled anatomical structures which is considered as gold standard. (C, E) The same MR images overlaid with the structural parcellation result after applying our multi-atlas framework. Top row: coronal view, bottom row: axial view.

For the highest average Dice similarity coefficient we obtained, the number of atlases for label fusion was equal to 8 and Gaussian kernel standard deviation was equal to 3 voxels. Sample images of the cross validation of our pipeline on the original atlas database as well as the atlas with left-right hemisphere separation are shown in Figure 3. This parameter combination was used to access the difference between our framework and two other approaches, as reported in the following section.

### Statistical Comparison

**Figure 4:**
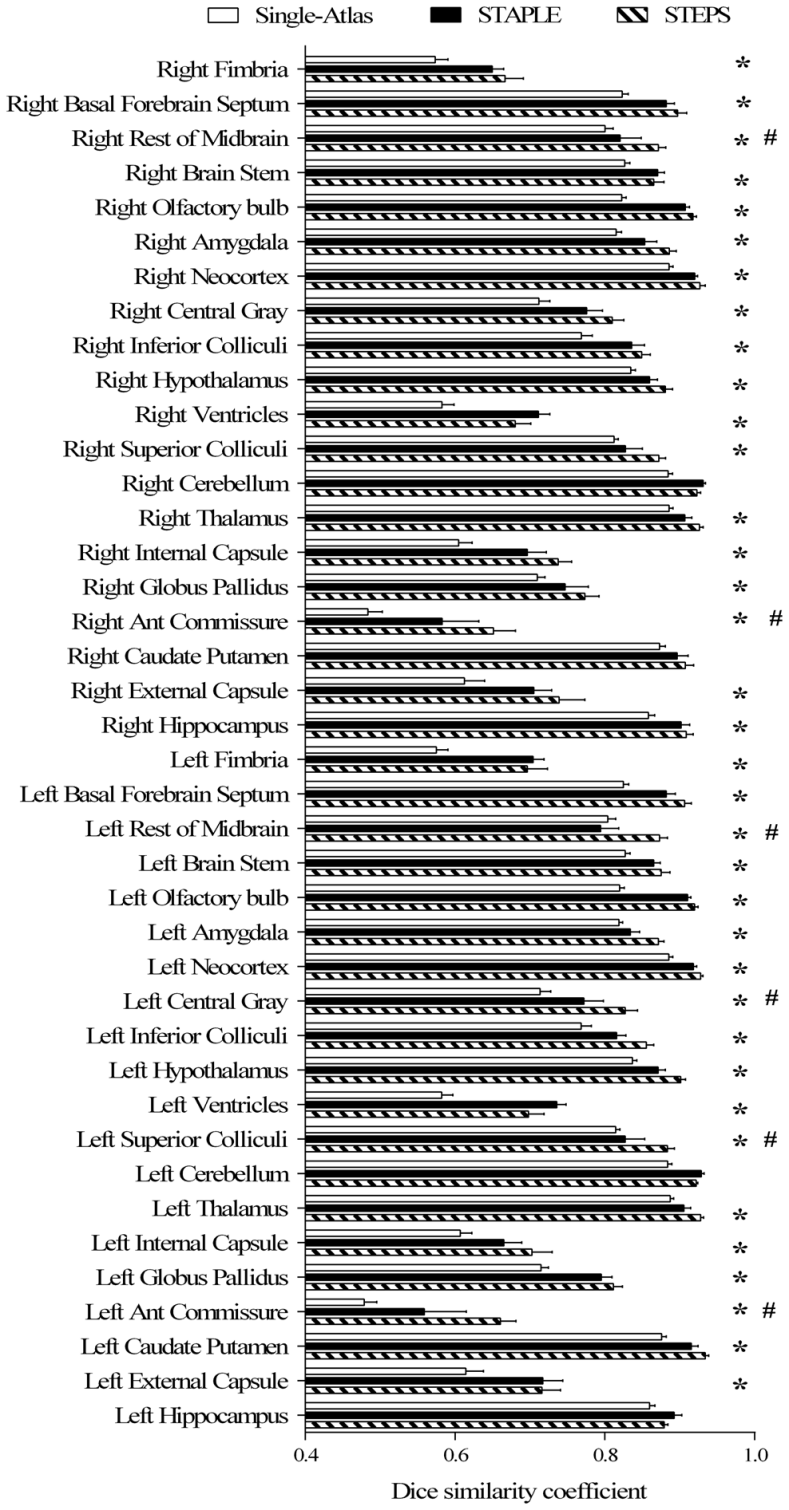
Cross validation result on the *in vivo* mouse brain atlas MRM NeAt [3]. Comparison of the average Dice similarity coefficient using our framework, a single-atlas segmentation propagation method and the STAPLE algorithm. Two-tailed paired t-tests were performed, with multiple comparisons of 40 structures corrected with false discovery rate set to 5%. Error bars representing standard deviation (*: significant difference was discovered between single-atlas method and STEPS algorithm; #: significant difference was discovered between STAPLE and STEPS algorithm).

We compared the parcellation accuracy of our framework with the single-atlas based segmentation propagation and STAPLE label fusion methods using a leave-one-out validation on the MRM NeAt atlas database. For each atlas image, we averaged the Dice similarity coefficient of all the propagated atlases. Both approaches were compared with the STEPS algorithm using an optimised parameter combination (Figure 4). A two-tailed paired t-test was performed on each of the 40 structures, and multiple comparisons across all the structures was corrected with FDR set to q = 0.05. Compared to the single-atlas method, our multi-atlas framework achieved significantly higher segmentation accuracy for every structures except the left hippocampus, left/right cerebellum, and right caudate putamen. Compared to the STAPLE algorithm, significantly higher segmentation accuracies are achieved in the left/right anterior commissure, left superior colliculi, left central gray and the remaining left/right midbrain olfactory bulb, brain stem and fimbria for both left and right hemispheres.

### Application to Unseen Images

**Figure 5:**
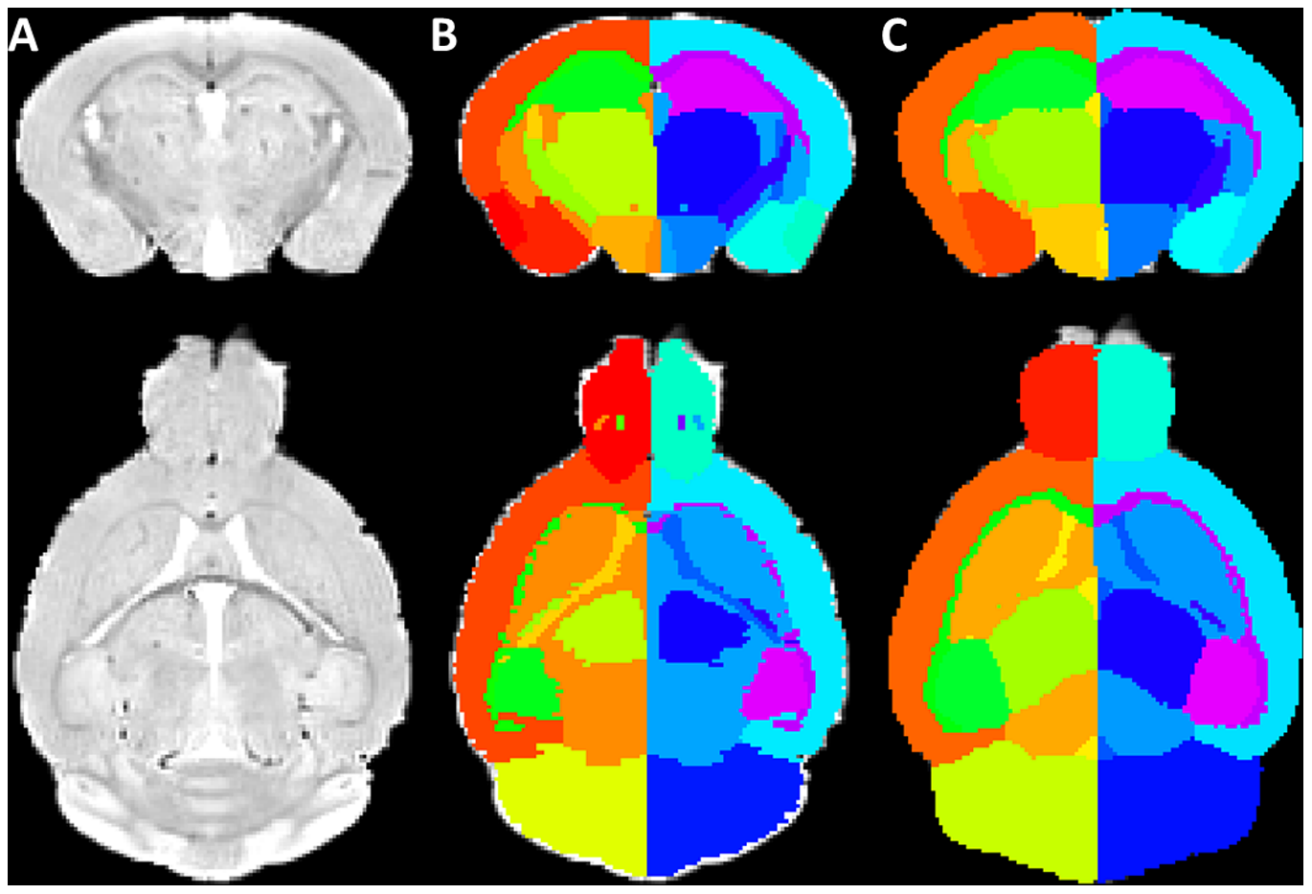
The structural parcellation result of applying our multi-atlas framework to a new dataset. (A) The MR image from an NUS mouse atlas which is treated as a new dataset. (B) MR image of the unlabelled image overlaid with corresponding manually labelled anatomical structures considered as gold standard. (C) The same MR images overlaid with the structural parcellation result after applying our multi-atlas framework. Top row: coronal view, bottom row: axial view.

**Figure 6:**
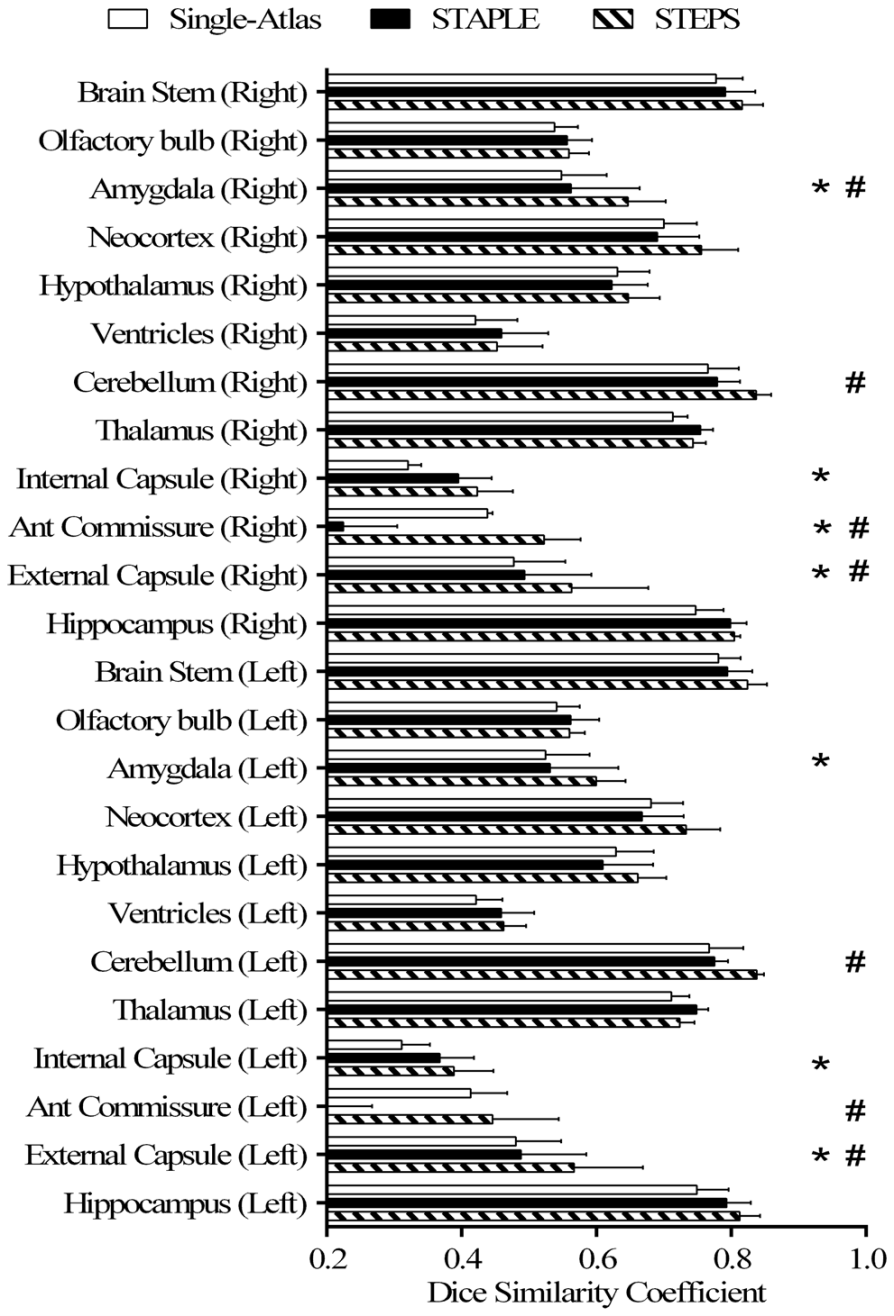
Validation on the ability of the multi-atlas framework to parcellate structures of the new dataset. The new dataset is adopted from the NUS mouse atlas [5] with the corresponding manual labels regarded as gold standard. 12 manually segmented structural labels were included in the comparison which appeared in both of the two atlas databases. Previously obtained optimised parameter combination for the MRM NeAt atlas database were used to calculate the Dice similarity coefficient. Two-tailed paired t-tests were performed, with multiple comparisons of 24 structures corrected with false discovery rate set to 0.05. Error bars representing standard deviation (*: significant difference was discovered between single-atlas method and STEPS algorithm; #: significant difference was discovered between STAPLE and STEPS algorithm).

In order to evaluate the ability of our multi-atlas framework to parcellate new data, we adopted the mouse brain MR images in the NUS atlas database as unlabelled test images. We propagated the structural labels in the MRM NeAt atlas database to the MR images in the NUS atlas database with the optimised parameter combination obtained previously for the MRM NeAt atlas database. Figure 5 shows the sample images of the test MRI data overlaid with corresponding manual labels as well as the automatic structural parcellation after applying our multi-atlas framework. The 24 manual structural labels (12 in each hemisphere) that were present in both atlas databases were selected and grouped. Figure 6 shows the statistical comparison of the resulting Dice similarity coefficient derived from our multi-atlas framework as well as that from the STAPLE algorithm and the single-atlas segmentation propagation. One should note that the Dice similarity may be of limited use due to the intrinsic variability between the manual segmentation protocols in the two atlas databases. The Dice similarity coefficient obtained here should neither be compared to that derived in the parameter optimisation part nor to the results from Bai et al’s study [5], because the images differ in contrast and SNR. A two-tailed paired t-test was performed on each of the 24 structures, with multiple comparisons across all the structures corrected with FDR (q = 0.05). When compared to the single-atlas method, our multi-atlas framework achieved significantly higher segmentation accuracy to parcellate left/right external capsule, left/right internal capsule, right anterior commissure, left olfactory bulb and right amygdala. Compared to the STAPLE algorithm, significantly higher differences are achieved in the external capsule, anterior commissure, cerebellum and neocortex for both hemispheres, and amygdala for right hemisphere. Interestingly, the performance of STAPLE is worse than the single atlas method when parcellating the anterior commissure. It could be due to the fact that the STAPLE algorithm assumes that the segmentation errors, for each individual segmentation, are due to random human rater error. However, a large portion of the segmentation errors here are due to image registration. The anterior commissure is a small structure, resulting in a relatively low impact on the registration algorithm compared to the contrast from surrounding tissues. On the other hand, the STEPS algorithm reduced the segmentation error coming from registration by taking the local image similarity into account in the atlas selection procedure [21].

### Application to Groupwise Analysis

**Figure 7:**
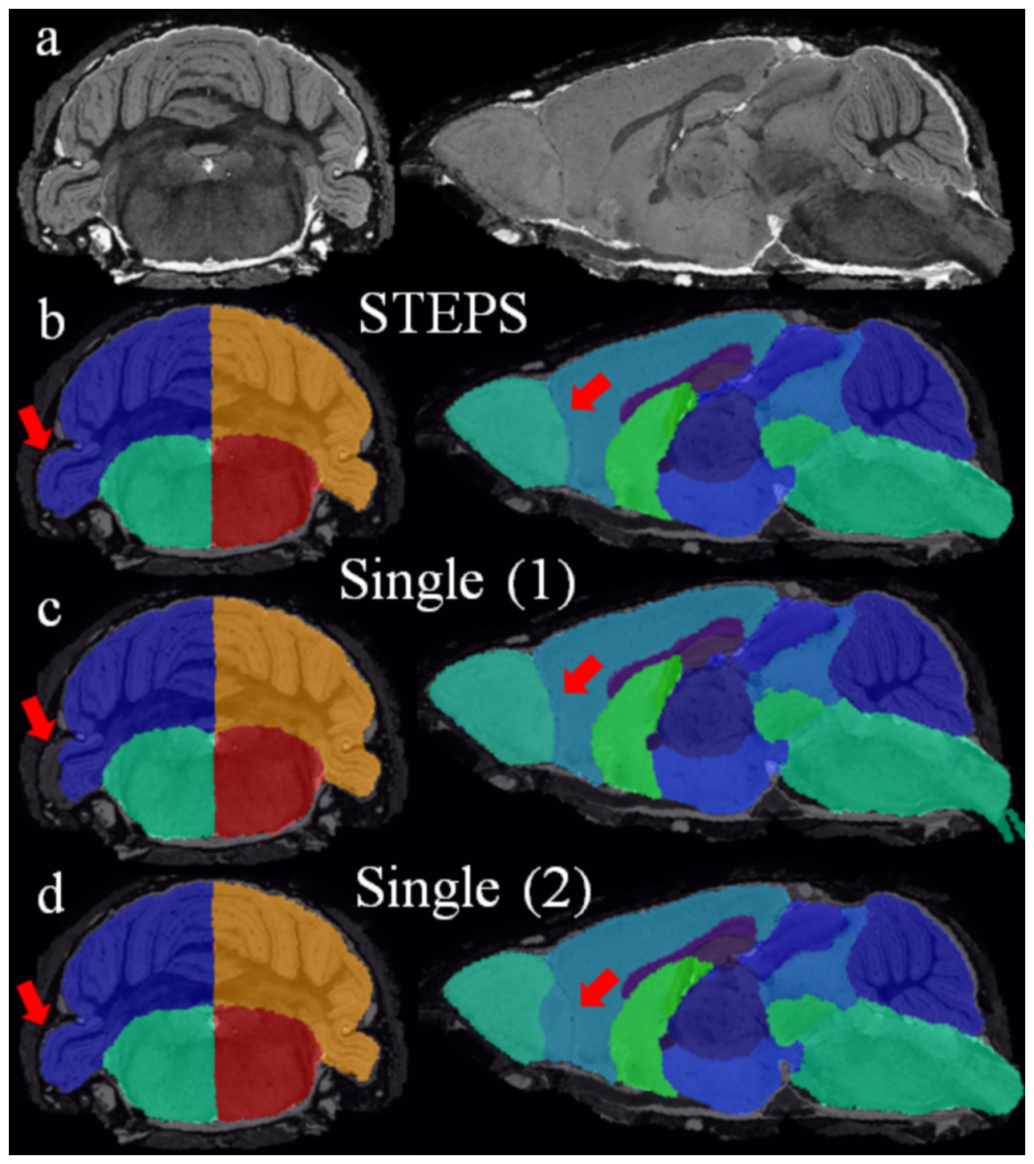
Sample images comparing the parcellation result of our framework and the single-atlas based methods. The selected slices demonstrated that despite some local misalignments in the single-atlas based method (as shown in red arrows). The STEPS label fusion algorithm in our framework successfully preserved the correct local registration in different regions. Structural parcellations are overlaid on the original image (in both coronal and sagittal view, a). (b) Structural parcellation using the proposed framework. (c) Structural parcellation result of a single-atlas based method with part of the cerebellum mis-segmented. (d) Another structural parcellation result of single-atlas based method with the edge between olfactory bulb and cortex mis-segmented.

**Figure 8:**
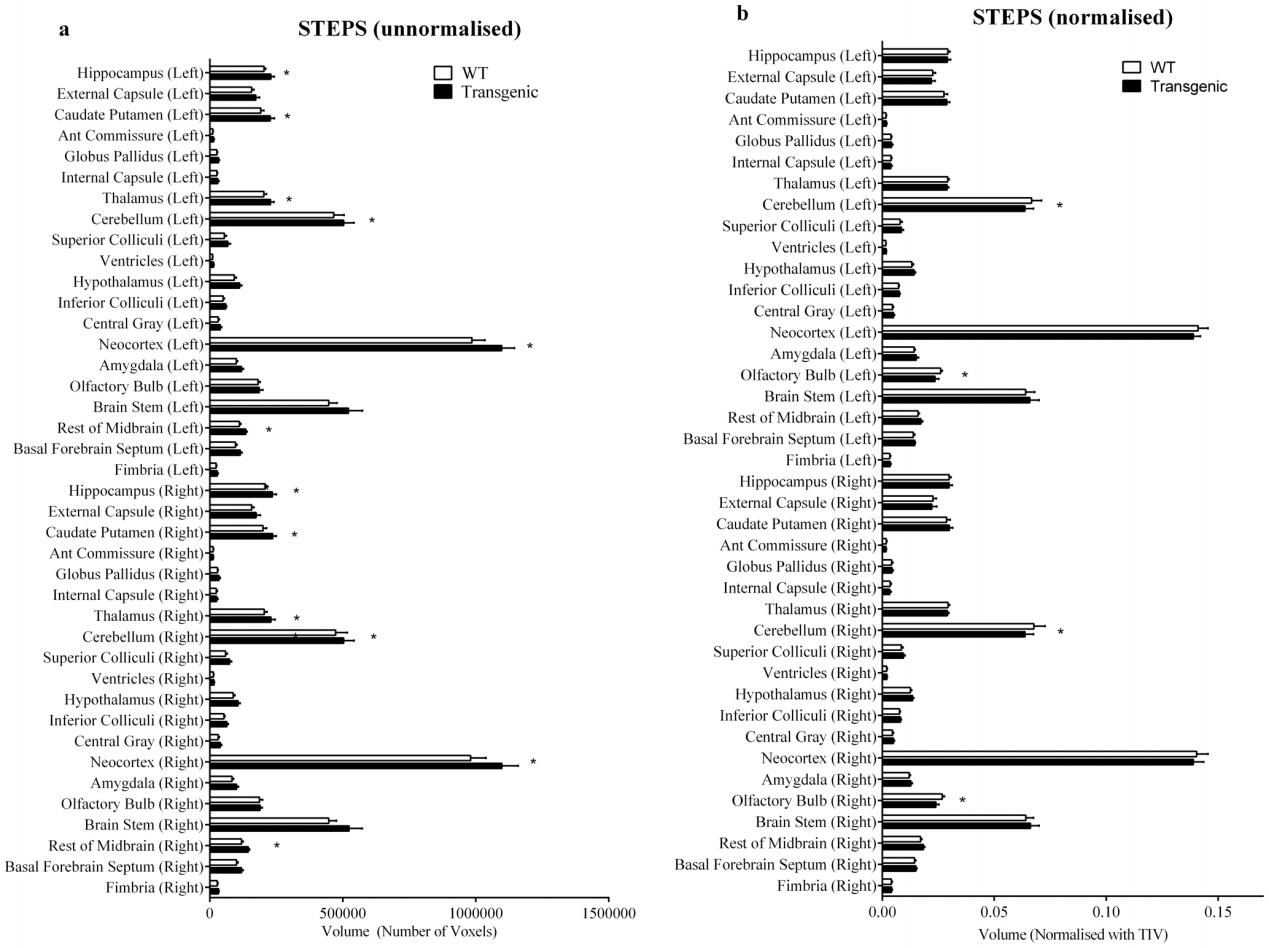
Statistical comparison of the structural volume difference between groups of Tc1 Down Syndrome mouse and wild type. Volumetric comparison on the a) unnormalised data; b) data normalised by total intracranial volume. A two-tailed paired t-test was performed on each of the 40 structures. Multiple comparisons are corrected with false discovery rate q = 0.05. Error bars representing standard deviation. (*: significant difference was discovered between the wild type and the transchromosomic group.).

Figure 7 shows sample images of parcellation results using our framework (Figure 7b) as well as using the single-atlas based method with misalignments occurring in some regions (Figure 7c, 7d). The STEPS label fusion algorithm successfully obtained the correct labels at both regions where not all of the single-atlas based methods produce accurate labels (shown by the red arrow). Statistical analysis between Tc1 Down Syndrome and wild type mouse was performed on the volumetric data both with and without total intracranial volume normalisation. A two-tailed paired t-test was performed on each of the 40 structures. Multiple comparisons were corrected with a false discovery rate q = 0.05 (Figure 8). We compared the statistical result of our framework with the result of the single-atlas based method for each of the 9 atlases in the database (Table 1).

**Table 1:** Statistical significant result on the volumetric comparison between groups of Tc1 Down Syndrome mouse and wild type, result obtained both from our multi-atlas framework as well as the single-atlas based method using all atlases in the database.

	Before normalisation										After normalisation									
Discovery?	STEPS	A1	A2	A3	A4	A5	A6	A7	A8	A9	STEPS	A1	A2	A3	A4	A5	A6	A7	A8	A9
Hippocampus (Left)	*		*	*				*		*										
External Capsule (Left)			*					*												
Caudate Putamen (Left)	*		*	*			*	*	*	*										
Ant Commissure (Left)																				
Globus Pallidus (Left)																				
Internal Capsule (Left)																				
Thalamus (Left)	*			*				*												
Cerebellum (Left)	*		*	*	*			*	*	*	*									
Superior Colliculi (Left)																				
Ventricles (Left)																				
Hypothalamus (Left)																				
Inferior Colliculi (Left)																				
Central Gray (Left)																				
Neocortex (Left)	*	*	*	*	*	*	*	*	*	*				*						*
Amygdala (Left)																				
Olfactory Bulb (Left)										*	*	*								
Brain Stem (Left)	*			*				*												
Rest of Midbrain (Left)																				
Basal Forebrain Septum (Left)																				
Fimbria (Left)	*		*	*				*												
Hippocampus (Right)			*																	
External Capsule (Right)	*		*	*	*		*	*	*	*										
Caudate Putamen (Right)																				
Ant Commissure (Right)																				
Globus Pallidus (Right)																				
Internal Capsule (Right)	*			*				*		*										
Thalamus (Right)	*		*	*				*	*	*	*	*		*	*					*
Cerebellum (Right)																				
Superior Colliculi (Right)																				
Ventricles (Right)																				
Hypothalamus (Right)																				
Inferior Colliculi (Right)																				
Central Gray (Right)	*	*	*	*	*	*	*	*	*	*										*
Neocortex (Right)																				
Amygdala (Right)										*	*	*			*					
Olfactory Bulb (Right)	*		*	*																
Brain Stem (Right)																				
Rest of Midbrain (Right)																				

STEPS: structural label obtained from the result of our framework using STEPS label fusion algorithm. A1–A9: structural label obtained from the result of single-atlas based segmentation method on each one of the atlas in the MRM NeAt database. A two-tailed paired t-test was performed on each of the 40 structures. Multiple comparisons are corrected with false discovery rate q = 0.05. Error bars representing standard deviation. (*: significant difference was discovered between the wild type and the transchromosomic group.).

For the unnormalised volumetric data, our framework detected significant volume increase in the transchromosomic group in hippocampus, caudate putamen, thalamus, cerebellum, neocortex and rest of the midbrain in both left and right hemispheres. Conversely, in the single-atlas method, five out of nine atlases (A1 A4 A5 A6 A8) failed to detect all significant volume increases as shown in our framework, one (A3) showed the same significant result, and one (A9) showed a significant result on the olfactory bulb which is neither detected by our framework nor the tensor-based morphometry analysis in the original study [49]. This was possibly due to a larger variance in the single-atlas based method. On the other hand, two atlases (A2 A7) showed a significant increase in external capsule which was not picked up by our framework, although they failed to detect significant differences either for the thalamus on the left hemisphere (A2) or for the rest of the midbrain on the right hemisphere (A7).

While for the volume normalised by the total intracranial volume, our framework detected significant volume shrinkage in the cerebellum and olfactory bulb that coincides with the tensor-based morphometry results reported by the original study, while all the single-atlas based methods detected less or no significant volume differences, suggesting less statistical power. It is worth noting that for the previously mentioned single-atlas based method on A2 and A7, which revealed additional statistical group difference on external capsule in the unnormalised data, they showed no statistical difference on all the structures after total intracranial volume normalisation.

Overall, our framework obtained better statistical power to detect structural group volume differences when compared to the single-atlas method. Nevertheless, some of the structural volume differences detected by the tensor-based morphometry analysis in the original study, such as superior colliculus and hypothalamus for the unnormalised data, and external capsule (posterior part of the corpus callosum) for the normalised data, were not captured using the proposed framework. This is possibly due to the voxel-wise nature of tensor-based morphometry techniques, which can detect very local changes, as opposed to the proposed technique, which can only detect changes in regional volume. Furthermore, an accurate structural parcellation is not only important for regional volume analysis, but also for further quantitative analyses such as thickness or shape analysis.

## Conclusions and Discussion

### Conclusion and Further Development

This paper presents a fully automatic multi-atlas framework for structural parcellation of mouse brain MRI data. The proposed work adopted a multi-atlas label fusion method – STEPS, along with an efficient non-rigid registration algorithm and other pre-processing techniques such as brain extraction and intensity non-uniformity correction using N3, to create an integrated framework for brain structural parcellation of mouse brain MR data.

Previous studies have shown successful applications of such multi-atlas segmentation propagation techniques in a clinical context, to detect volumetric variation of brain structures such as ventricles and hippocampi [9], [20]. In this study, the results denote that with a relatively limited number of available atlases in the database when compared with clinical studies, the structural parcellation of pre-clinical data can be significantly improved when compared to previous approaches [12], [13]. We have also demonstrated the ability of our framework to use existing atlas databases to parcellate images acquired at different sites. We also tested its ability to detect brain structure volumetric changes in genetically modified pathological animal model.

In order to assess the pre-clinical relevance of the proposed framework, further work could include statistical power analysis. For example, would the improved method be able to detect the same amount of change by using fewer samples, or what level of subtle change can be detected using the new method with the same amount of data? van Eede et al. have recently proposed a method to generate artificial deformation field which was originally used to test registration sensitivity [50], which would be a good method to generate simulated volume changes.

### Parameter Optimisation Related Issues

The optimised STEPS parameters were chosen based on the average Dice similarity coefficient over all the structures and across all samples in the atlas database. However, one should note that the Dice similarity coefficient is intrinsically biased towards large structures (e.g. hippocampus and neocortex), while small structures (e.g. external capsule, anterior commissure) are more sensitive to local registration errors and inter-atlas morphological variation. However, studies interested in parcellating only certain structures can obtain the optimised parameter combinations following this framework by considering only the Dice similarity coefficient for the specific structures of interest.

When dealing with new data, there is likely to be no manual segmentation associated with the data. As a result, it is impossible to improve the parameters for the pipeline further in order to reach the underlying optimal parcellation. However, as shown in Figure 2, the parameter optimisations near the optimal value reach a plateau. It indicates that our pipeline is resistant to parameter variation around the optimal combination. As a result, small deviations of the parameter values have a small impact on the overall segmentation performance. On the other hand, when applying the pipeline to a set of unseen data from another study, the parcellation error derived from image registration would increase. Within the two parameters of interest, the “Kernel standard deviation” of LNCC- is directly related to the image registration error, while the number of selected atlas is more related to the statistical power of the label fusion. It is shown in Figure 2 that the parameter “Kernel standard deviation” has less effect in the segmentation accuracy when compared to the number of selected atlases. We speculate thus that the optimised parameters obtained through the leave-one-out validation will not be too distant from the underlying optimal parameter when applying to the new data.

### Image Registration Related Issues

Most image similarity measurements used in registration algorithms are governed by high contrast edges, and the registration accuracy in regions with low contrast is limited. For the neighbouring anatomical regions that lack contrast in between, the registration algorithm will have to rely on the regularisation term rather than on image features for accurate matching [31], [51]. This can lead to a decrease in segmentation performance. In addition, the atlases used for the proposed multi-atlas framework are limited in number and are T2* weighted, which might impede their direct application to images acquired with different contrast. However, the normalised mutual information used in this framework for image similarity measurement has been shown to be less dependent on image contrast, and is currently commonly used to compare image similarity between multi-modal images [52].

### Current Limitation of Mouse Brain Study

Compared to human brain MRI segmentation studies [14], [20], [21], the availability of mouse brain atlas databases is lacking, and as such, the performance of label fusion techniques is subsequently limited. To the best of our knowledge, there are currently only two *in vivo* multi-atlas mouse brain MRI databases that are publicly available [3], [5]. The number of available databases, as well as the number of atlases in each database, is far from ideal. Although Chakravarty et al. [27] improved the segmentation accuracy by introducing an artificial intermediary multi-atlas database from a single-atlas, it does not address the problem of insufficient data and morphometric variability.

It has been shown that label fusion algorithms benefit from an increase in atlases, as the statistical power increases with sample size [8]. The brain has similar structural layout across two hemispheres. Studies have shown that by including the flipped mirror images of the atlases to double the database size, the structural parcellation result can be improved [20], [21], [45], [46]. This might arguably be an alternative solution to the problem of limited atlas numbers in the database. However, when testing on the MRM NeAt atlas database, the improvement of such process is limited (data shown in supplementary material “File S1. Mirroring process”). This might be due to the small number of atlases available in the database, which reduces the chance to get better local morphological match from the flipped images for the label fusion algorithm to benefit from, while additional registration error is introduced at other regions due to the brain asymmetry. Furthermore, MRI has been used to measure the asymmetry of adult mouse brains [53], and a recent study using optogenetics conducted by Michael et al. also showed differences between the left and right hippocampal plasticity [54]. Given such lateralization of the brain, further validation is still necessary to assess the anatomical viability of such flipping process.

Within the field of clinical research, there are well documented and standardised protocol of manual parcellation [55]–[57], the amount of equivalent available information for mouse brain MRI is however limited. The unclear nature of the anatomical standardisation and vague definitions of the segmentation protocol also reduces consistency between human raters. Furthermore, manual segmentations are considered the gold standard to evaluate segmentation accuracy, and are used for comparison to assess the performance of automatic segmentation methods. This makes the intra- and inter-rater labelling variability crucially important as it represents the theoretical performance upper limit for an automatic method. Such variability has not been fully assessed in mice, which makes it difficult to determine the potential improvement that an algorithm can achieve. Most of the available publications about mouse brain MRI atlas construction, either *in vivo* or *ex vivo*, single-atlas based or multi-atlas based, lack clear guidance about the protocol for manual segmentation [3], [26], [34], [58]. However, efforts are being made to address this. Bai et al. included a detailed protocol for manual segmentation of every structure that is parcellated in the *in vivo* atlas they released in the supplementary material [5]. More recently, Ullmann et al. from the Australian Mouse Brain Mapping Consortium described a detailed segmentation protocol on the minimal deformation atlas for *ex vivo* MR images on the C57BL/6J mouse, which provided further information for segmenting sub-regions of the neocortex, hippocampus, cerebellum, and basal ganglia which would be a good guideline for future investigations [4], [39], [40], [59]. Those studies will eventually lead to a standardised consensus protocol for manual segmentation of mouse brain MRI.

## Supporting Information

File S1 **Mirroring process.** This supporting information describes the process and experimental result of including the flipped mirroring images of the atlases to double the database size. This might arguably be an alternative solution to the limited atlas number in the database, and have been shown to improve the structural parcellation result [20], [21], [45], [46].
(DOC)

File S2 **MRI mouse brain atlas databases currently available.** This supporting information contains detailed description and comparison of the 7 publicly available atlas database which are mentioned in the “Mouse brain atlas” section of the manuscript [3], [5], [26], [34]–[40].
(DOC)
