# Supplementary material for: Automatic Structural Parcellation of Mouse Brain MRI Using Multi-Atlas Label Fusion
Source: PLoS One. 2014 Jan 27;9(1):e86576. doi: 10.1371/journal.pone.0086576 (PMC3903537; doi:10.1371/journal.pone.0086576)
Supplement: File S1 — Mirroring process. This supporting information describes the process and experimental result of including the flipped mirroring images of the atlases to double the database size. This might arguably be an alternative solution to the limited atlas number in the database, and have been shown to improve the structural parcellation result [20], [21], [45], [46]. (DOC) [file pone.0086576.s001.doc]

# Supplementary – Mirroring process

# Background

We follow the idea of several previous study on clinical MRI data [1–4], and double the database size by including a left/tight flipped version of all the atlases. The purpose of this test is to see whether the accuracy of the structural parcellation can be improved through such mirroring process. The hypothesis is that the local atlas selection algorithm LNCC could benefit from the atlas doubling, and the final parcellation accuracy (in terms of Dice similarity coefficient) can be increased.

# Methods

We investigated the segmentation accuracy of our multi-atlas framework by applying it to the original left/right hemisphere separated atlas database as well as to the database including the mirrored atlases, with the optimal parameter combination for each database. The segmentation accuracy was presented in terms of Dice similarity coefficient for each structure (Figure 1). A two-tailed paired t-test was performed on each of the 40 structures. Multiple comparison across all the structures is corrected with False Discovery Rate (FDR) set to q=0.05.

## Results

After multiple comparison is corrected, significant difference remains for the fimbria and ventricles in the right hemisphere only (Figure 1). This result shows that the improvement of the including flipped version of the atlases is not obvious. More detailed discussion about this result is shown in the main manuscript section 4.4.

## Figures

**Figure 1: Comparison of atlas database with or without flipped atlases.**

**
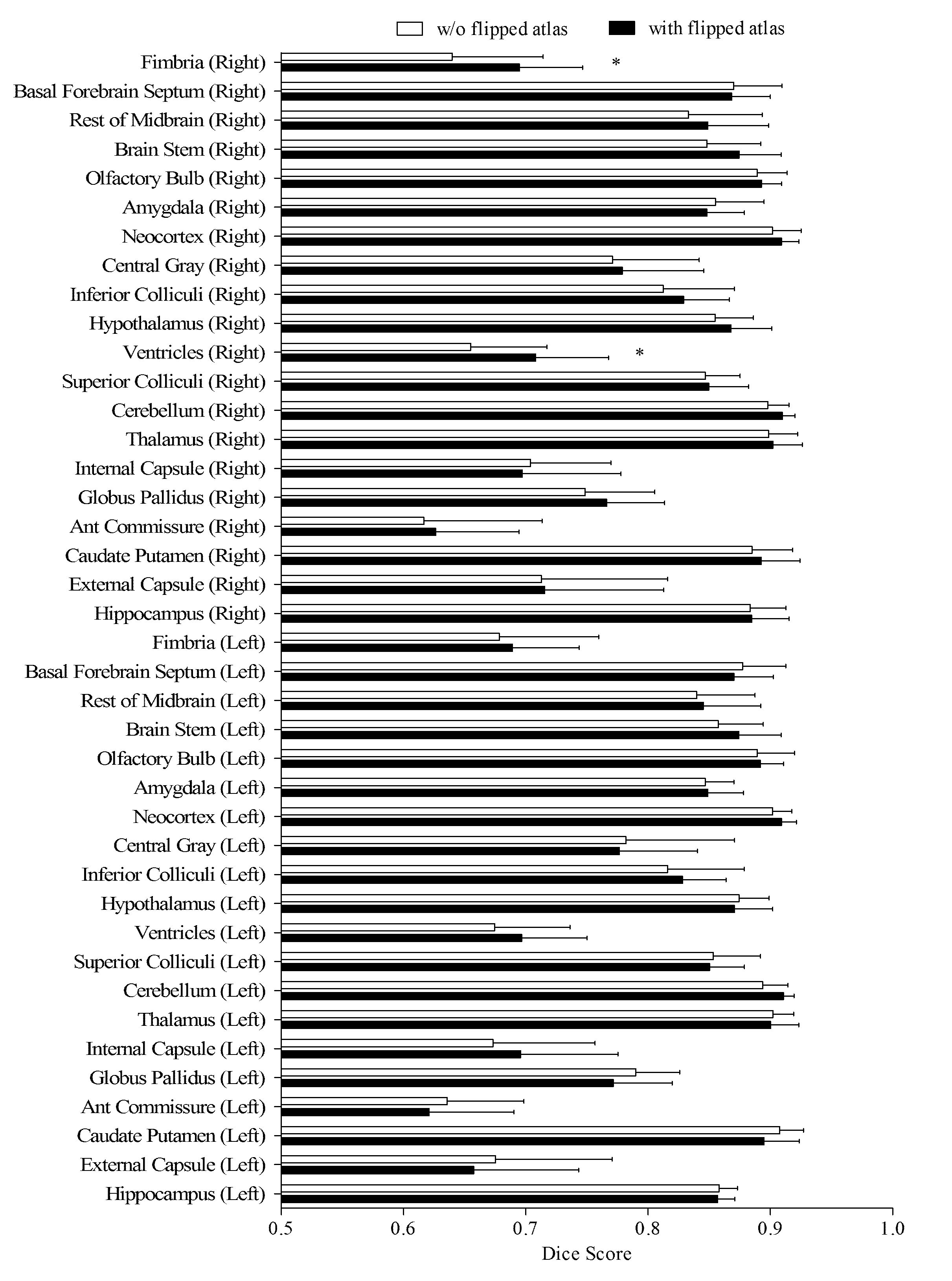
**

Dice similarity coefficients of each structures were obtained by applying the framework to the database with only left/right hemisphere separated atlases and the database with mirrored atlases, using the optimal parameter combination for each database. Two-tailed paired t-tests were performed, with multiple comparison of 40 structures corrected with false discovery rate set to 5%, Error bars representing standard deviation (*: significant difference was detected between two groups).

## Reference

1. Leung KK, Barnes J, Ridgway GR, Bartlett JW, Clarkson MJ, et al. (2010) Automated cross-sectional and longitudinal hippocampal volume measurement in mild cognitive impairment and Alzheimer’s disease. Neuroimage 51: 1345–1359.

2. Cardoso M, Leung K, Modat M, Keihaninejad S, Cash D, et al. (2013) STEPS: Similarity and Truth Estimation for Propagated Segmentations and its application to hippocampal segmentation and brain parcelation. Med Image Anal.

3. Wang H, Suh JW, Das S, Pluta J, Altinay M, et al. (2011) Regression-Based Label Fusion for Multi-Atlas Segmentation. Conf Comput Vis Pattern Recognit Work IEEE Comput Soc Conf Comput Vis Pattern Recognit Work 20: 1113–1120.

4. Wang H, Suh JW, Das SR, Pluta JB, Craige C, et al. (2013) Multi-Atlas Segmentation with Joint Label Fusion. Pattern Anal Mach Intell IEEE Trans 35: 611–623.
