# Supplementary material for: Automatic Structural Parcellation of Mouse Brain MRI Using Multi-Atlas Label Fusion
Source: PLoS One. 2014 Jan 27;9(1):e86576. doi: 10.1371/journal.pone.0086576 (PMC3903537; doi:10.1371/journal.pone.0086576)
Supplement: File S2 — MRI mouse brain atlas databases currently available. This supporting information contains detailed description and comparison of the 7 publicly available atlas database which are mentioned in the “Mouse brain atlas” section of the manuscript [3], [5], [26], [34]–[40]. (DOC) [file pone.0086576.s002.doc]

# Supplementary - Online Mouse Brain MRI Atlas Database (up to Oct, 2013)

|  | Mouse Image Centre, Toronto Centre for Phenogenomics | John Hopkins University School of Medicine | Mouse Atlas Project 2.0, Laboratory of Neuro Imaging, UCLA  Mouse Connectome | Centre for in vivo microscopy, Duke University Medical Centre | Neurological Atlas Mouse Brain Database, University of Florida | National University of Singapore | Australia mouse brain mapping consortium |
| --- | --- | --- | --- | --- | --- | --- | --- |
| Mouse type | Neuroanatomy : 12 weeks C57B1/6J + 8 week 129S1/Svlmj  Variational: 129SV/S1 | C57BL/6 (E12-18,P0-P80 and older) | Neonatal C57BL/6J (p0);  100 days C57BL/6J | 9-12 weeks C57BL/6J | 12-14 week C57BL/6J | Adult C57BL/6J | 12 weeks male C57BL/6J |
| Imaging type | T2W | T2W + DTI (FA, DEC^1^) | T2W (neonatal);DWI (adult) | T1, T2W, T2*, DTI | T2* *ex vivo* / *in vivo* | T2W | T1/T2*-weighted |
| Atlas type | Average atlas along with corresponding labels | Averaged atlas; | Along z-direction, label volume, label index; | Averaged atlas | Individual atlas, average atlase, probabilistic atlas and average voxel deformation maps | Individual atlas | Average atlas |
| Sample number | *Neuroanatomy*: 62 structure, 40 samples;  *Variational*: 40 labels, 9 samples | Major gray/white structures; Embryonic (E12-E18)+postnatal (P7,11,15,21,28,42,60); 6 samples for each time point | *Neonatal*: 13 structure (by averaging brain map/ probability maps threshold);  *Adult*: not mentioned | 9 white matter structure;  8 samples | *Ex vivo*: 20 structures,10 samples  *In vivo*: 20 structures,12 samples | *In vivo*: 40 structures, 5 samples | *Ex vivo*: 38 cerebellum subregions 74 cortical subregions, |
| Scanning parameter | 7T (Magnex+Varian) | 11.7T (Bruker) | 11.7T (Bruker) | 9.4T (GE) | *Ex vivo*: 17.6T (U Florida +Bruker)  *In vivo*: 9.4T (Magnex+Bruker) | *In vivo*: 7T (Bruker) | *Ex vivo*: 16.4T (Bruker) |
| Resolution | C57B1/6J: 32 μm  129S1/Svlmj : 54 μm | *Embryo*: 80 μm  *Adult*: 125 μm | *Neonatal*: 50×70×70 μm  *Adult*: 60 μm | 43 μm (voxel volume ~80 pl) | *Ex vivo*: 47 μm  *In vivo*: 100 μm | *In vivo*: 100 μm | *Ex vivo*: 30 μm |
| Availability | Open access | Open access (registration needed)^2^ | Open access(registration need) | Open access | Open access (need registration) | Open access | Open access |
| Website | <http://www.mouseimaging.ca/technologies/mouse_atlas.html> | <http://lbam.med.jhmi.edu/> | [map.loni.ucla.edu](http://map.loni.ucla.edu/)  [www.birncommunity.org/data-catalog/mouse-3d-mr-minimum-deformation-atlas/](http://www.birncommunity.org/data-catalog/mouse-3d-mr-minimum-deformation-atlas/) | [http://www.civm.duhs.duke.edu](http://www.civm.duhs.duke.edu/neuroYJ201009) | <http://www.bnl.gov/ctn/mouse>  <http://phenome.jax.org/> | <http://www.bioeng.nus.edu.sg/cfa/atlas/mouse.html> | [www.imaging.org.au/AMBMC/AMBMC](http://www.imaging.org.au/AMBMC/AMBMC) |
| Publication | [1–3] | [4,5] | [6] | [7,8] | [9,10] | [11] | [12–15] |
| Additional information | Other atlas type in the database: Cerebral Vascular Atlas of the CBA Mouse; Also include vascular atlas for developing mouse embryo | Other atlas type in the database: white matter tractional map | Other atlas type in the database: mouse Connectome Project;  MBAT (Mouse BIRN Atlas Toolkit); Rat atlas.  Franklin-Paxinos space | Also with rat atlas available | Collaborator: Brookhaven National Laboratory; Stony Brook State University of New York.  Part of the MPD project: Mouse Phenome Database |  |  |

# Other atlases:

**Caltech μMRI Atlas of Mouse Development**:

[http://mouseatlas.caltech.edu](http://mouseatlas.caltech.edu/) (embryo)

**Allen Institute for Brain Science & European mirror: INCF (International Neuroinformatics Coordinating Facility) online comprehensive atlas of the mouse brain**:

<http://www.brain-map.org/>; <http://www.incf.org/programs/atlasing> (histological atlas, including: connectivity/diversity/developing mouse brain atlas)

**Montreal Neurological Institute, Brain Imaging Centre**

[http://www.bic.mni.mcgill.ca](http://www.bic.mni.mcgill.ca/) BrainWeb (human)

# Reference:

1. Chan E, Kovacevíc N, Ho SKY, Henkelman RM, Henderson JT (2007) Development of a high resolution three-dimensional surgical atlas of the murine head for strains 129S1/SvImJ and C57Bl/6J using magnetic resonance imaging and micro-computed tomography. Neuroscience 144: 604–615.

2. Dorr AE, Lerch JP, Spring S, Kabani N, Henkelman RM (2008) High resolution three-dimensional brain atlas using an average magnetic resonance image of 40 adult C57Bl/6J mice. Neuroimage 42: 60–69.

3. Kovacević N, Henderson JT, Chan E, Lifshitz N, Bishop J, et al. (2005) A three-dimensional MRI atlas of the mouse brain with estimates of the average and variability. Cereb Cortex 15: 639–645.

4. Chuang N, Mori S, Yamamoto A, Jiang H, Ye X, et al. (2011) An MRI-based atlas and database of the developing mouse brain. Neuroimage 54: 80–89.

5. Aggarwal M, Zhang J, Mori S (2011) Magnetic resonance imaging-based mouse brain atlas and its applications. Magn Reson Neuroimaging: 251–253.

6. MacKenzie-Graham A, Lee E-F, Dinov ID, Bota M, Shattuck DW, et al. (2004) A multimodal, multidimensional atlas of the C57BL/6J mouse brain. J Anat 204: 93–102.

7. Jiang Y, Johnson GA (2011) Microscopic diffusion tensor atlas of the mouse brain. Neuroimage 56: 1235–1243.

8. Badea A, Ali-Sharief AA, Johnson GA (2007) Morphometric analysis of the C57BL/6J mouse brain. Neuroimage 37: 683–693.

9. Ma Y, Hof PR, Grant SC, Blackband SJ, Bennett R, et al. (2005) A three-dimensional digital atlas database of the adult C57BL/6J mouse brain by magnetic resonance microscopy. Neuroscience 135: 1203–1215.

10. Ma Y, Smith D, Hof PR, Foerster B, Hamilton S, et al. (2008) In Vivo 3D Digital Atlas Database of the Adult C57BL/6J Mouse Brain by Magnetic Resonance Microscopy. Front Neuroanat 2: 1.

11. Bai J, Trinh TLH, Chuang K-H, Qiu A (2012) Atlas-based automatic mouse brain image segmentation revisited: model complexity vs. image registration. Magn Reson Imaging 30: 789–798.

12. Ullmann JFP, Keller MD, Watson C, Janke AL, Kurniawan ND, et al. (2012) Segmentation of the C57BL/6J mouse cerebellum in magnetic resonance images. Neuroimage 62: 1408–1414.

13. Ullmann JFP, Watson C, Janke AL, Kurniawan ND, Reutens DC (2013) A segmentation protocol and MRI atlas of the C57BL/6J mouse neocortex. Neuroimage 78: 196–203.

14. Ullmann JP, Watson C, Janke A, Kurniawan N, Paxinos G, et al. (2013) An MRI atlas of the mouse basal ganglia. Brain Struct Funct: 1–11.

15. Richards K, Watson C, Buckley RF, Kurniawan ND, Yang Z, et al. (2011) Segmentation of the mouse hippocampal formation in magnetic resonance images. Neuroimage 58: 732–740.
